# Solution-Processed Hybrid Light-Emitting Devices Comprising TiO_2_ Nanorods and WO_3_ Layers as Carrier-Transporting Layers

**DOI:** 10.1186/s11671-016-1733-x

**Published:** 2016-11-24

**Authors:** Tsung-Yan Tsai, Po-Ruei Yan, Sheng-Hsiung Yang

**Affiliations:** Institute of Lighting and Energy Photonics, National Chiao Tung University, No. 301, Gaofa 3rd Road, Guiren District, Tainan, 71150 Taiwan, Republic of China

**Keywords:** Titanium dioxide nanorods, Tungsten trioxide, Inverted light-emitting devices, Electron-transporting layers

## Abstract

The goal of this research is to prepare inverted light-emitting devices with improved performance by combining titanium dioxide (TiO_2_) nanorods and tungsten trioxide (WO_3_) layer. TiO_2_ nanorods with different lengths were established directly on the fluorine-doped tin oxide (FTO) substrates by the hydrothermal method. The prepared TiO_2_ nanorods with lengths shorter than 200 nm possess transmittance higher than 80% in the visible range. Inverted light-emitting devices with the configuration of FTO/TiO_2_ nanorods/ionic PF/MEH-PPV/PEDOT:PSS/WO_3_/Au were constructed. The best device based on 100-nm-height TiO_2_ nanorods achieved a max brightness of 4493 cd/m^2^ and current efficiency of 0.66 cd/A, revealing much higher performance compared with those using TiO_2_ compact layer or nanorods with longer lengths as electron-transporting layers.

## Background

Organic light-emitting devices (OLEDs) have made impressive progress during the past two decades due to high luminance efficiency, wide viewing angle, flexibility, and low cost [1, 2]. They also show highly potential applications in the areas of displays and solid-state lighting. Despite those advantages, there still exist many challenges for organic light-emitting devices to replace liquid crystal displays and inorganic light-emitting diodes due to shorter lifetime at high luminescence and sensitivity to oxidation and humidity.

Conventional light-emitting devices are usually constructed with the configuration of anode/hole-transporting layer (HTL)/active layer/electron-transporting layer (ETL)/metal cathode. Poly(3,4-ethylenedioxythiophene): poly(styrene sulfonate) (PEDOT:PSS) is the most used material as HTL. However, the acidic nature of PEDOT:PSS can lead to the corrosion of the oxide anode; moreover, high-efficiency devices usually require highly active metals as the cathode, e.g., calcium or magnesium, which are easy to oxidize. In view of the above reasons, inverted light-emitting devices have been proposed and drawn increasing attention mainly due to higher stability compared with conventional ones. The usage of high work function metals such as gold (Au) or silver (Ag) as anode and air-stable metal oxide materials as carrier-transporting layers consequently prolongs lifetime of inverted-type devices. The architecture of inverted light-emitting devices is constructed with the configuration of cathode/ETL/active layer/HTL/metal anode [3, 4]. Many *n*-type metal oxide materials, including zinc oxide (ZnO), titanium dioxide (TiO_2_), and tin dioxide have been used as ETL [5–7]. On the other hand, *p*-type materials such as vanadium oxide, nickel oxide, and tungsten trioxide (WO_3_) can be utilized as HTL [8–10]. Taking TiO_2_ as an example, it is a well-known semiconducting material that possesses high thermal stability, good electron-transporting property, large energy bandgap of 3.0–3.4 eV, and friendly to our living environment [11]. TiO_2_ is normally a white solid; however, it can be transparent in thin film or in some nanostructured states. By changing the morphology of TiO_2_, it is expected that the optical and/or electrical transporting properties of TiO_2_ are also modified. In fact, nanostructured TiO_2_ have been applied in many areas, such as ultraviolet photodetectors [12], OLEDs [13], organic solar cells [14], and photocatalytic water-splitting technology [15]. Morii et al. reported encapsulation-free hybrid organic/inorganic light-emitting diodes with the configuration of fluorine-doped tin oxide (FTO)/TiO_2_/F8BT/MoO_3_/Au [16], using TiO_2_ as an electron injection layer (EIL) and MoO_3_ as a hole injection layer (HIL). The device could be operated in air with a lower threshold voltage that provided similar luminance output compared with conventional devices using Ca/Al as cathode. Bolink et al. reported inverted light-emitting devices with the configuration of indium tin oxide (ITO)/ZnO/Cs_2_CO_3_/super yellow PPV/MoO_3_/Au [17], using ZnO as an EIL and Cs_2_CO_3_ as a hole-blocking layer. High luminance and current efficiency of 12,000 cd/m^2^ and 6.5 cd/A were achieved that is comparable with conventional devices. Park et al. proposed inverted light-emitting devices with the configuration of ITO/ZnO/Cs_2_CO_3_/F8BT/PEDOT:PSS/Ag [18]. The Cs_2_CO_3_ layer was spin-cast into a thin film from its solution, not by thermal evaporation. A max luminance of 3399 cd/m^2^ and max current efficiency of 0.81 cd/A around 14–16 V were obtained. Song et al. further modified a device structure with the configuration of FTO/ZnO/FPQ-Br/F8BT/MoO_3_/Au [19], using an ionic polyfluorene (PF) derivative FPQ-Br tethering Br^−^ counterions as the wetting agent to improve contact between inorganic ZnO and organic F8BT. Wen et al. employed a self-assembled monolayer PEDA-TMS to modify TiO_2_ surface for tuning its conduction band to match the energy level of high-yellow phenyl-substituted poly(*para*-phenylenevinylene) copolymer (HY-PPV). Inverted devices with the configuration of ITO/TiO_2_/PEDA-TMS/HY-PPV/MoO_3_/Au/Ag were fabricated, revealing much higher brightness of 3148 cd/m^2^ than that of the bare TiO_2_ device [20]. From literature survey, we notice that the above metal oxide materials were prepared and utilized as a thin film; TiO_2_ nanorods has not been reported in the light-emitting area so far. In addition to TiO_2_ and ZnO thin films, TiO_2_ nanorods are regarded as a potential candidate for the construction of light-emitting devices based on the following reasons. As mentioned in the previous part, TiO_2_ is a cheap, thermally stable, and non-toxic semiconducting material that is favorable for device fabrication and mass production. Moreover, nanorod-type TiO_2_ can provide one-dimensional pathways for carrier transportation. This is particularly beneficial for electrons to enter the device and to improve recombination rate of carriers. The transmittance of TiO_2_ in the visible region can also be tuned by experimental controls. From a scientific and industrial viewpoint, developing alternatives or new type of materials is an essential issue to expand the diversity of research instead of adopting limited materials.

**Fig. 1:**
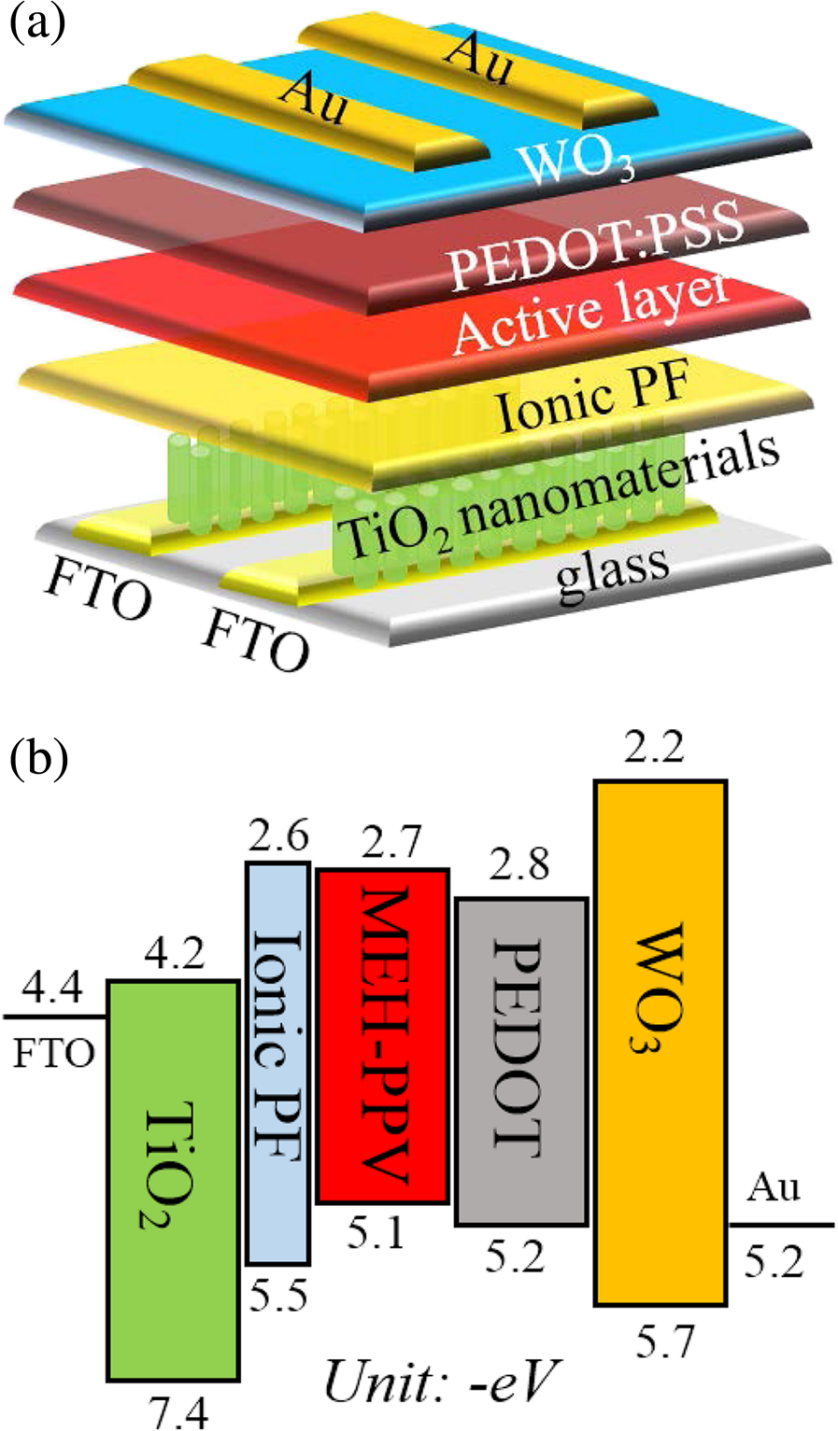
**a** Device architecture and **b** energy level diagram of the whole device

In this research, we demonstrate the preparation and characterization of TiO_2_ nanomaterials, including nanoparticles, nanorods, and compact layer, which can be used as an ETL for the fabrication of hybrid-inverted light-emitting devices. TiO_2_ nanorods and nanoparticles were prepared on the FTO substrates by the hydrothermal method without using either templates or seeds. This is because the FTO substrate also has the tetragonal rutile structure, and the lattice mismatch between the tetragonal FTO (*a* = *b* = 0.4687 nm) and the rutile TiO_2_ (*a* = *b* = 0.4593 nm) is only 2% [21]. To further improve device performance, ultra-thin layers of tungsten trioxide (WO_3_) and ionic PF derivative are incorporated. WO_3_ has been reported to serve as the HIL for application in organic light-emitting and photovoltaic devices [22–24]. The ionic PF material carrying hexafluorophosphate (PF_6_^−^) counterions as wetting agent was synthesized and demonstrated in this study. Inverted light-emitting devices with the configuration of FTO/TiO_2_ nanorods/ionic PF/MEH-PPV/PEDOT:PSS/WO_3_/Au were fabricated and evaluated. The illustration of the device structure is shown in Fig. 1a, and the energy level diagram of the whole device is illustrated in Fig. 1b. It is seen that electron injection from FTO to TiO_2_ layer is undisturbed [25]. To overcome the large energy barrier between TiO_2_ and the active layer MEH-PPV, a thin layer of ionic PF was introduced; besides, this ionic PF also serves as the wetting layer to increase contact between inorganic TiO_2_ and organic MEH-PPV layers. On the other hand, the valence band of WO_3_ lies between the highest occupied molecular orbital of PEDOT:PSS and the work function of gold electrode that is favored for hole injection from the anode [22]. The PEDOT:PSS layer is incorporated between WO_3_ and the active layer to increase hole transfer. The recombination of electrons and holes in the active layer MEH-PPV results in electroluminescence (EL) under bias operation. The features of inverted architecture and solution process for deposition of organic and inorganic layers in this study provide a promising way to low-cost manufacturing in the future.

## Methods

### Polymer Materials

**Fig. 2:**
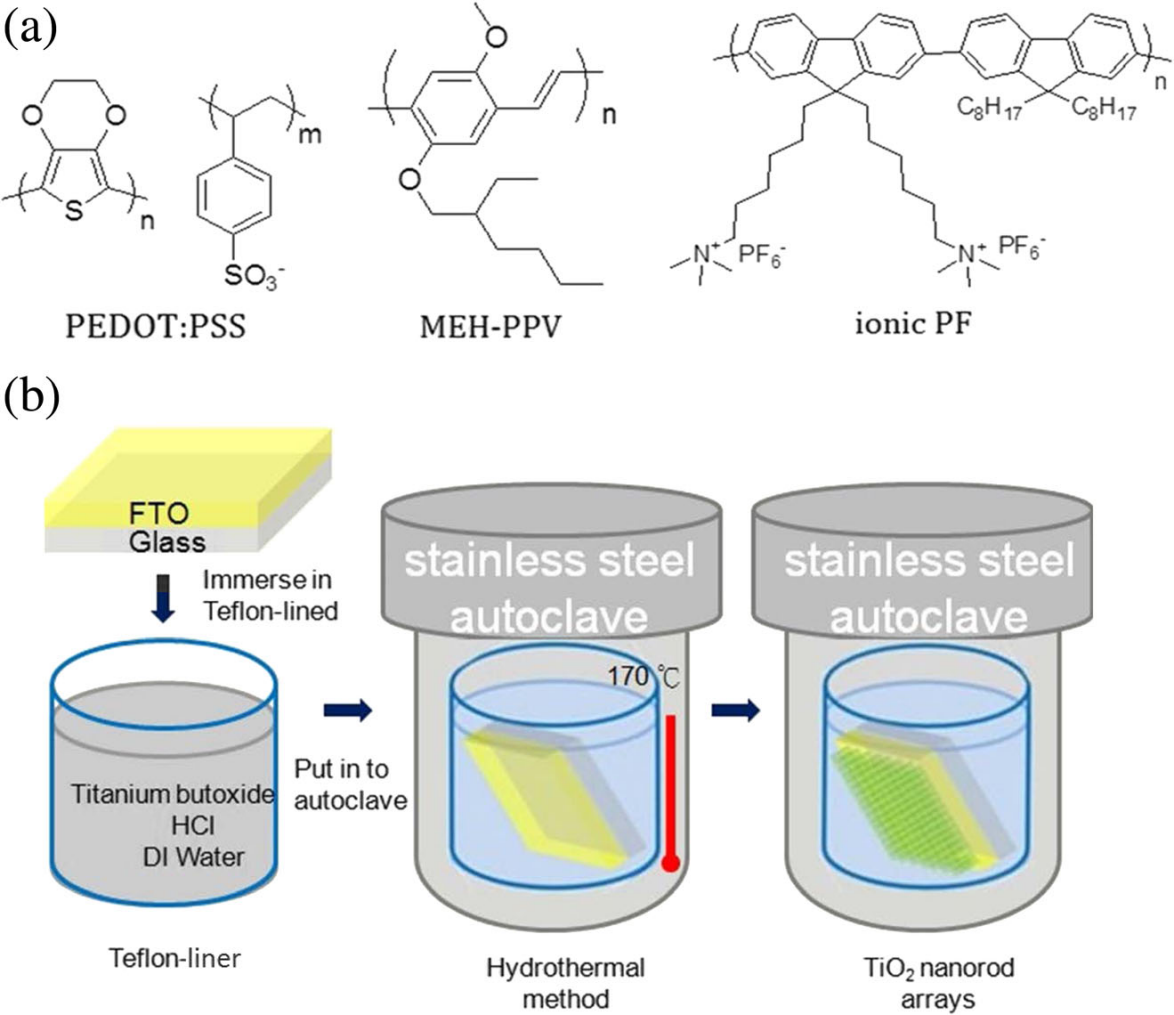
**a** Chemical structures of organic polymers used in this study; **b** schematic illustration of growth process of TiO_2_ nanorods on the FTO substrate

The light-emitting polymer poly(2-methoxy-5-(2′-ethylhexyloxy)-1,4- phenylenevinylene) (MEH-PPV) was synthesized according to the literature [26]. PEDOT:PSS aqueous solution (CleviosTM P VP AI 4083) was purchased from Heraeus Precious Metals GmbH & Co. KG. Titanium(IV) *n*-butoxide and Titanium(IV) chloride was purchased from Alfa. Ammonium hexafluorophosphate was purchased from Matrix Scientific. Hydrochloric acid was purchased from ECHO Chemical. The solvents including methanol, acetonitrile, toluene, and ethanol were also purchased from ECHO Chemical. These reagents and solvents were used as received without further purification. The ionic PF carrying PF_6_^−^ groups was synthesized by ionic exchange from its precursor PF-Br according to the previous literatures [27]. The detailed synthetic procedure of this ionic PF is described as follows. To a solution of PF-Br (100 mg) in methanol (20 mL) was slowly added a solution of ammonium hexafluorophosphate (0.4 g, 4.8 mmol) in methanol (20 mL). The mixture was stirred at room temperature for 48 h, followed by removing the solvent by rotary evaporation. The previous procedure was repeated for 4 or 5 times to achieve high percentage of ionic exchange from Br^−^ to PF_6_^−^. The final product was collected and dried in an oven to give a yellow solid (90 mg, 75%). The chemical structures of the above materials are shown in Fig. 2a.

### Preparation of TiO_2_ Nanomaterials

The schematic illustration of the growth of TiO_2_ nanoparticles and nanorods on FTO substrates by the hydrothermal method [28] is shown in Fig. 2b, and the detailed preparation is described as follows. Thirty-seven percent Hydrochloric acid (20 mL) was added in 20 mL of deionized water and stirred for 10 min. Titanium(IV) *n*-butoxide (0.7 mL) was then added and stirred for an additional 10 min. The solution mixture was transferred into the autoclave in which the FTO substrates were placed at an angle against the wall of the teflon-liner. The autoclave was placed in a preheated oven at 170 °C. The growth time was set to 100, 110, 120, and 130 min. TiO_2_ nanoparticles were formed with the growth time of 100 min, while nanorods with lengths of 100, 200, and 300 nm were obtained with the growth time of 110, 120, and 130 min, respectively. The substrates were taken out, rinsed thoroughly with deionized water, and calcined at 450 °C in air for 1 h. The TiO_2_ compact layer was also prepared via the chemical bath deposition for comparison in this study [29]. A solution of 1 M TiCl_4_ in toluene (1 mL) was added in 4 mL of deionized water dropwise at 0 °C and stirred for 5 min. The FTO substrates were then immersed into this solution and placed in an oven at 70 °C for 1 h. The substrates were taken out, washed with deionized water and ethanol, and calcined at 450 °C in air for 1 h.

### Fabrication of Inverted Light-Emitting Devices

Inverted light-emitting devices with the configuration of FTO/TiO_2_ nanomaterials/ionic PF/MEH-PPV/PEDOT:PSS/WO_3_/Au were fabricated. The device architecture is shown in Fig. 1a. The ionic PF in acetonitrile (1 mg/1 mL) was spin-coated on top of the TiO_2_ layer, followed by drying in a vacuum oven at 90 °C for 30 min. MEH-PPV film (150 nm) was spin-cast from its toluene solution (15 mg/1 mL) and dried in a vacuum oven at 90 °C for 30 min. PEDOT:PSS layer (50 nm) was spin-cast on top of MEH-PPV and baked in a vacuum oven at 90 °C for 30 min. WO_3_ layer (5 nm) was prepared by spin-coating from its precursor solution (1 mg of W(OEt)_5_ in 1 mL of anhydrous ethanol) and placed in ambient environment for 15 min. Finally, Au electrodes were deposited by thermal evaporation at a base pressure of 10^−6^ torr. The active area of each device is 4 mm^2^.

### Characterization Methods

The transmission and absorption spectra of TiO_2_ nanomaterials were measured with a Princeton Instruments Acton 2150 spectrophotometer. The surface morphology of the materials was studied using the tapping-mode Bruker Innova atomic force microscopy (AFM). Cross-sectional scanning electron microscopy (SEM) micrographs of devices were performed with a JEOL 6700 F SEM. X-ray diffraction (XRD) patterns were obtained from a Rigaku D/MAX2500 diffractometer. The electrical and emission characteristics of light-emitting devices were measured using an Agilent 4155C semiconductor analyzer and a calibrated silicon photodiode.

## Results and Discussion

### Morphological Studies of TiO_2_ Nanomaterials

**Fig. 3:**
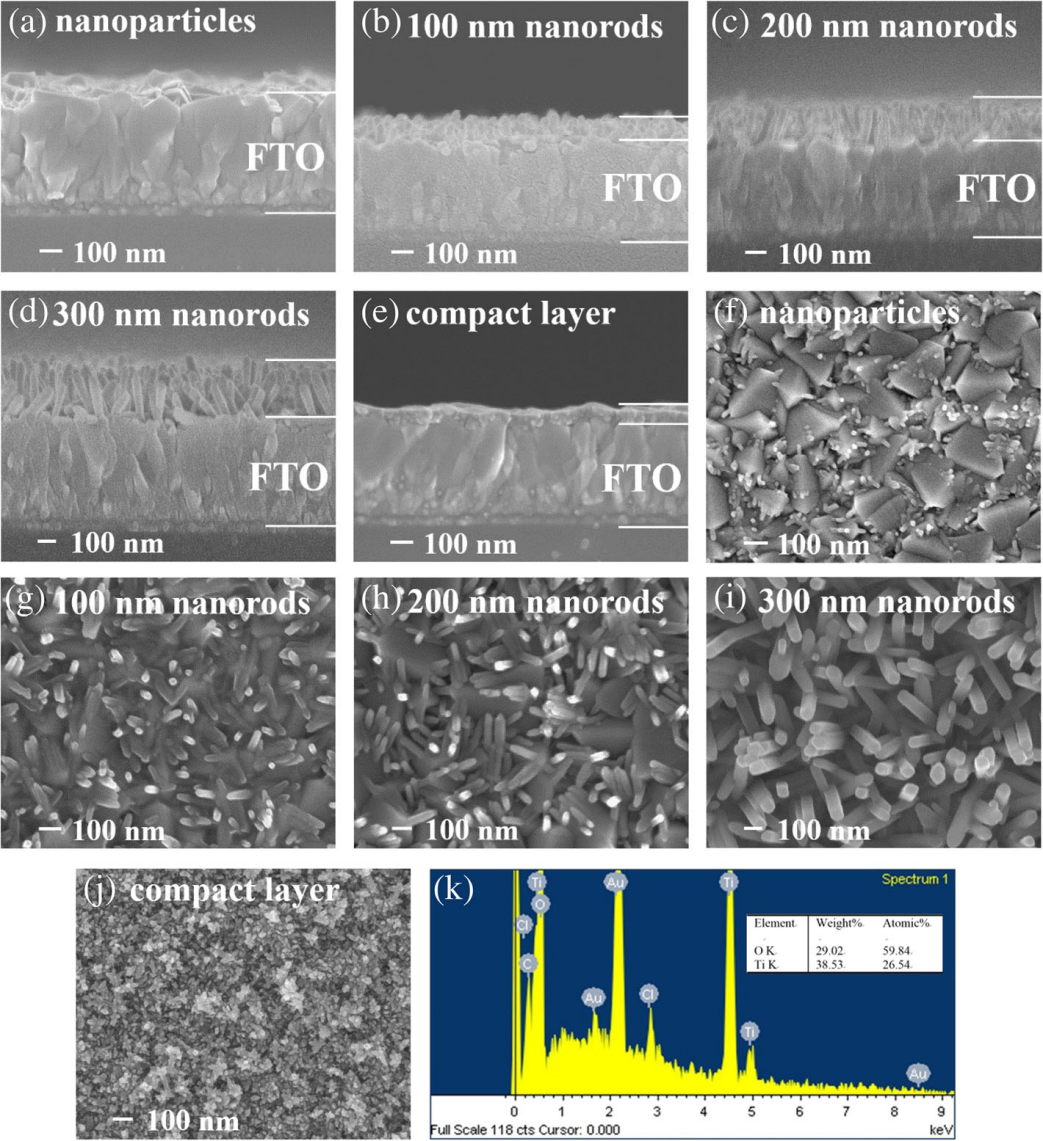
Side view SEM images of TiO_2_ **a** nanoparticles, **b** 100, **c** 200, and **d** 300-nm-height nanorods, and **e** compact layer; top view SEM images of TiO_2_ **f** nanoparticles, **g** 100, **h** 200, and **i** 300-nm-height nanorods and **j** compact layer; **k** EDX spectrum of 300-nm-height TiO_2_ nanorods

**Fig. 4:**
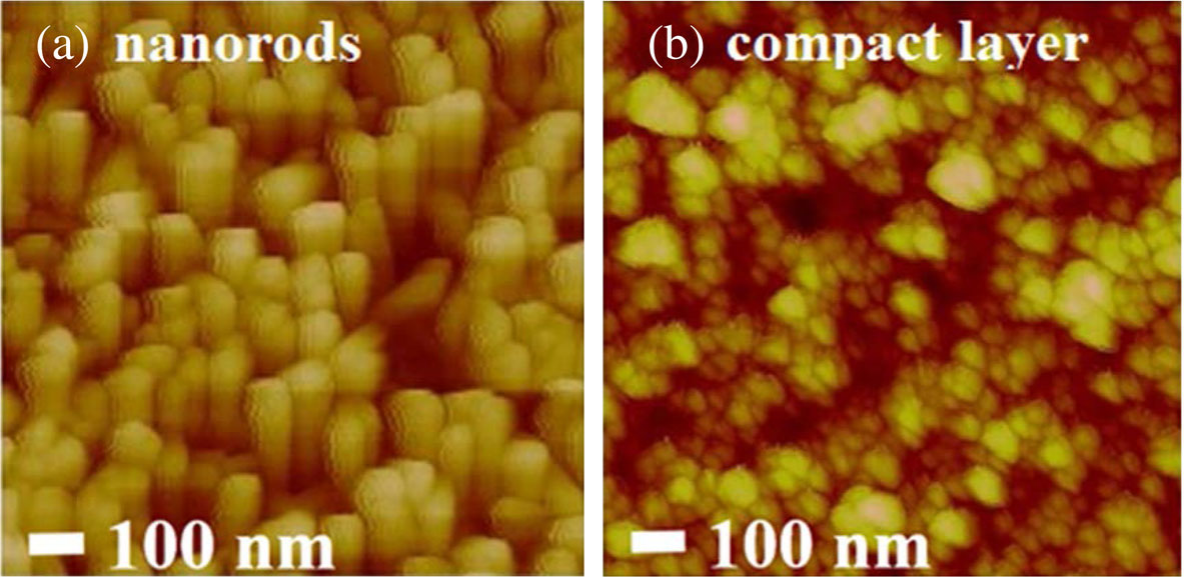
AFM topographic images of TiO_2_ **a** nanorods and **b** compact layer

The SEM micrographs of TiO_2_ nanomaterials on the FTO substrates are shown in Fig. 3. TiO_2_ nanoparticles under growth time of 100 min are sparsely dispersed on the surface of FTO, and the diameters of those TiO_2_ nanoparticles are estimated to be 20–30 nm, as verified in Fig. 3a and f. The growth and dispersion of TiO_2_ nanoparticles on the FTO is caused by the homogeneous nucleation of TiO_2_ [30, 31]. When the growth time prolongs to 110 min, TiO_2_ nanoparticles can serve as the seeds for further growth of nanorods as well as the heterogeneous nucleation of TiO_2_. It is seen that TiO_2_ nanorods are mainly grown upwards, with some nanorods tilt on the FTO substrates. From the SEM side view in Fig. 3b–d, the lengths of TiO_2_ nanorods are estimated to be 100, 200, and 300 nm when the growth time is set to 110, 120, and 130 min, respectively. From the SEM top view in Fig. 3g-i, the diameters of TiO_2_ nanorods is determined in the range of 40–80 nm. The lengths and diameters are concluded to be dependent on the growth time [25], i.e., nanorods grow longer and wider as the growth time increases. Besides, the FTO substrates are not fully covered by TiO_2_ nanoparticles or nanorods when the growth time is set shorter than 120 min. The SEM side view and top view images of TiO_2_ compact layer are shown in Fig. 3e and j, respectively. Large amounts of crystalline nanoparticles with total thickness of 100 nm are clearly observed for TiO_2_ compact layer, which is very different from those nanorods. The chemical stoichiometry of Ti and O atoms in the nanorods is examined with an energy dispersive X-ray spectroscopy (EDX) analyzer and shown in Fig. 3k. It indicates the presence of Ti and O with an atomic ratio of 1:2.25 that is in agreement with the stoichiometric composition of TiO_2_ [30]. The total surface area of TiO_2_ nanorods are supposed to be larger than the compact layer due to high aspect ratio of nanorods, which might show positive effect on carrier injection and transportation. Due to different surface properties (TiO_2_ is hydrophilic and organic materials are hydrophobic), an ionic PF was introduced between TiO_2_ and organic layer to serve as the wetting layer. The AFM topographic images of TiO_2_ nanorods and compact layer are shown in Fig. 4. Rod-like and particle-like morphologies are observed for TiO_2_ nanorods and compact layer, with surface roughness (*R*_*a*_) of 26.8 and 17.7 nm, respectively.

### Optical Properties of TiO_2_ Nanomaterials

**Fig. 5:**
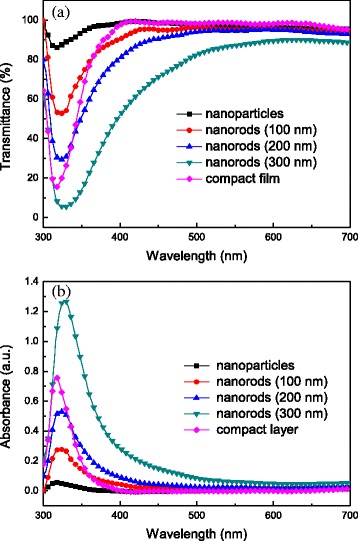
**a** Transmission and **b** absorption spectra of TiO_2_ nanoparticles, nanorods with a height of 100–300 nm, and compact layer

The transmission and absorption spectra of TiO_2_ nanomaterials on the FTO substrates were measured and shown in Fig. 5. The transmittance of TiO_2_ nanorods drops sharply in the range of 330–380 nm, arising from the nature of TiO_2_ absorption. For TiO_2_ nanoparticles, compact layer, and nanorods with lengths of 100 and 200 nm, the transmittance reaches over 80% in the visible range of 380–700 nm. As for TiO_2_ nanorods with a length of 300 nm, the transmittance is significantly lower than others due to longer height of nanorods and full coverage of FTO substrate. Nevertheless, moderate to high transparent property of TiO_2_ nanomaterials is suitable for optoelectronic applications. The main absorption band of TiO_2_ is observed from 300 to 380 nm. The absorption edges of TiO_2_ nanorods and compact layer are found at 400 and 380 nm, respectively. The band gap of the TiO_2_ nanorods and compact layer were then calculated from their absorption edges to be were 3.10 eV and 3.25 eV, respectively, which is in good accordance with the previous reports [32].

### XRD Patterns of TiO_2_ Nanomaterials

**Fig. 6:**
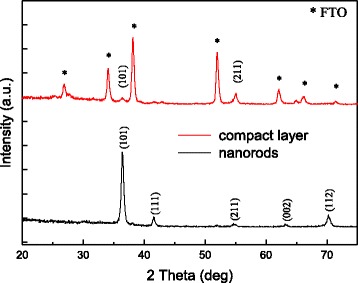
XRD patterns of 300-nm-height TiO_2_ nanorods and 100-nm-thick compact layer

The XRD patterns of the 300-nm-height TiO_2_ nanorods and 100-nm-thick compact layer on FTO substrate is presented in Fig. 6a, which is in good accordance with that of the rutile phase (JCPDS No. 88–1175) [30]. These diffraction peaks are sharp and strong, indicating high-degree crystallization of the prepared TiO_2_ nanomaterials in this research. The five main diffraction peaks located at *2θ* = 36.07°, 41.24°, 54.33°, 62.75°, and 29.3° are assigned to (101), (111), (211), (002), and (112) planes, respectively [30]. The highly intense (101) peak along with the enhanced (002) peak in the nanorods reveals that the rutile crystal grows with (101) plane parallel to the FTO substrate, and the nanorods are oriented along the (002) direction [33]. The diffraction peaks from the FTO substrates are also indicated in Fig. 6. Only two diffraction peaks assigning to (101) and (211) planes can be observed for TiO_2_ compact layer. The difference in XRD intensity between TiO_2_ nanorods and compact layer arises from different thickness of the layer. Besides, the mobility of rutile TiO_2_ nanorods is reported to be 1 cm^2^/Vs, which is two-order higher than that of TiO_2_ nanoparticles layer [33, 34]. The higher electron-transporting properties brought by the nanorod form are beneficial for device performance.

### Device Performance Based on TiO_2_ Nanomaterials

**Fig. 7:**
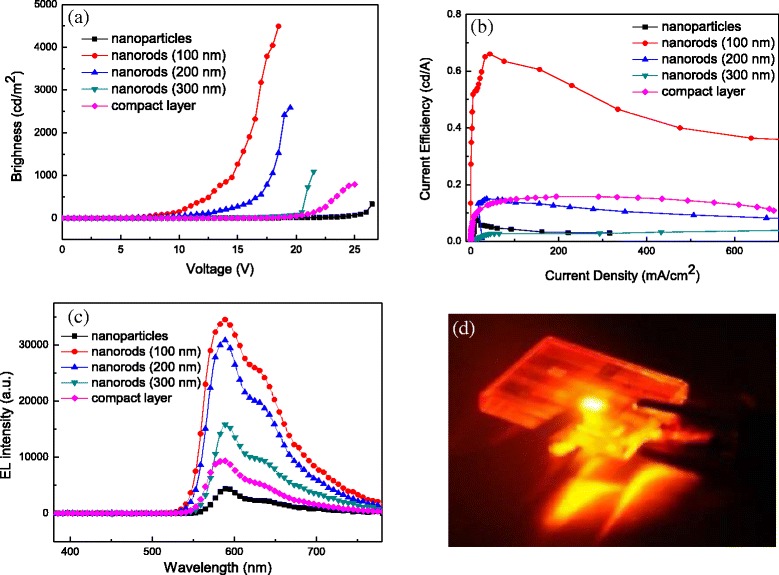
**a** B-V, **b** E-J, and **c** EL spectra of inverted light-emitting devices based on TiO_2_ nanoparticles, nanorods with a height of 100–300 nm, and compact layer. **d** Snapshot of the device based on 100-nm-height TiO_2_ nanorods driven at 15 V

**Fig. 8:**
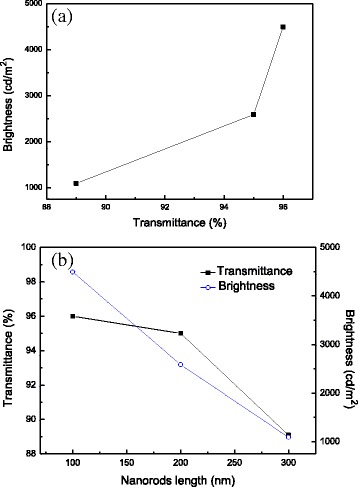
**a** Relationship between transmittance of TiO_2_ nanorods and brightness of devices; **b** characteristics of transmittance of TiO_2_ nanorods and brightness of devices as a function of TiO_2_ nanorod length

**Fig. 9:**
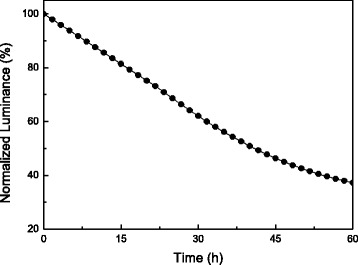
Luminance decay curve of the device based on 100-nm-height TiO_2_ nanorods as a function of time

Inverted light-emitting devices with the configuration of FTO/TiO_2_/ionic PF/MEH-PPV/PEDOT:PSS/WO_3_/Au were fabricated and evaluated, using TiO_2_ as electron-transporting layer, ionic PF as wetting layer, MEH-PPV as active layer, PEDOT:PSS as hole-transporting layer, and WO_3_ as hole-injecting layer. The brightness-voltage (B-V), current efficiency-current density (E-J) characteristics, and EL spectra of all devices are depicted in Fig. 7a–c. The overall device performance based on different types of TiO_2_ is summarized in Table 1. The max brightness and current efficiency of the device based on TiO_2_ nanoparticles reached 333 cd/m^2^ and 0.08 cd/A, respectively. By using TiO_2_ compact layer as an ETL, the device performance was promoted with max brightness of 790 cd/m^2^ and current efficiency of 0.15 cd/A. The reason to the improved performance is due to full coverage of FTO surface by the TiO_2_ compact layer that reduces charge trapping and quenching on FTO anode. Turing to TiO_2_ nanorods, the max brightness achieved 4493, 2589, and 1090 cd/m^2^ for the devices based on 100, 200, and 300-nm-height nanorods, respectively. Furthermore, the current efficiency was decreased from 0.66 to 0.05 cd/A as the length of TiO_2_ nanorods was increased. The reason to this phenomenon can be explained as follows. First, TiO_2_ nanorods with length of 100 nm own the highest transmittance among three lengths of nanorods, which is beneficial for light output. Second, 100-nm TiO_2_ nanorods provide shorter pathways for carrier injection to the active layer to generate light. We also notice that the devices based on TiO_2_ nanorods revealed much better device performance than others using TiO_2_ nanoparticles or compact layer as an ETL. This is due to higher mobility of TiO_2_ nanorods that is favored for carrier transport than the other two TiO_2_ nanomaterials, as described in the previous section. Figure 7c shows the original EL spectra of the inverted devices based on TiO_2_ nanomaterials operated at 12 V. The emission maximum wavelength and shoulder emission are located at 588 and 630 nm, respectively, revealing an orange-red light. A very bright inverted light-emitting device based on 100-nm-height TiO_2_ nanorods under driving bias of 15 V is shown in Fig. 7d. Figure 8a shows the relationship between the transmittance of TiO_2_ nanorods and the brightness of devices. The value of transmittance was chosen at 588 nm, since the device emitted light at the same wavelength. It is seen that TiO_2_ nanorods with higher transmittance bring higher brightness. To clarify the relationship between nanorod length and transmittance/brightness, the characteristics transmittance-nanorods and length-brightness are depicted and shown in Fig. 8b. As the nanorod length is increased, both transmittance and brightness are decreased. It is noted that the transmittance as well as the brightness of the devices in the whole visible region should be taken into account to completely understand the transmittance effect on the brightness of the devices, not only at 588 nm. Figure 9 shows the luminance decay curve as a function of time for the device based on 100-nm-height TiO_2_ nanorods. The device was monitored at a constant voltage of 10 V in ambient environment without encapsulation. The lifetime of the device is defined as the time when the luminance is decreased to a half of its initial luminance, which is determined about 40 h. The stability test of inverted OLEDs is seldom reported in the literature. G. He et al. reported the inverted OLEDs with the configuration of ITO/Cs_2_CO_3_/Bphen/Alq3/NPB/MoO_3_/Au [35]. The device lifetime was only 20 h when using Cs_2_CO_3_ as the EIL. By inserting a thin layer of aluminum between ITO and Cs_2_CO_3_, the luminance of the device decayed to 80% of its initial value during an operation time of 70 h. In our case, the usage of TiO_2_ nanorods as the ETL can provide moderate stability in ambient condition. To the best of our knowledge, this is the first demonstration of inverted light-emitting devices using TiO_2_ nanorods as ETL. These results suggest that TiO_2_ nanorods may possess potential use in light-emitting applications.

**Table 1:** Performance of inverted light-emitting devices based on TiO_2_ nanomaterials

TiO_2_ type	Turn-on voltage (V)	Max brightness (cd/m^2^)	Max current efficiency (cd/A)
Compact layer	14	790	0.15
Nanoparticles	13.5	333	0.08
Nanorods (100 nm)	4.5	4493	0.66
Nanorods (200 nm)	5.0	2589	0.15
Nanorods (300 nm)	9.5	1090	0.05

## Conclusions

TiO_2_ nanoparticles with diameter of 20–30 nm and nanorods with different lengths of 100–300 nm were prepared by the hydrothermal method. TiO_2_ compact layer was prepared by chemical bath deposition for comparison. The results showed that rutile TiO_2_ was obtained, and nanorods with shorter length of 100–200 nm showed higher transmittance in the visible range of 380–700 nm. Inverted light-emitting devices using different TiO_2_ nanomaterials as ETL were fabricated and compared. The device based on 100-nm-height TiO_2_ nanorods revealed the best device performance among all devices, with a max brightness and max current efficiency of 4493 cd/m^2^ and 0.66 cd/A, respectively.

## Abbreviations

AFM: Atomic force microscopy
B-V: Brightness-voltage
EDX: Energy dispersive X-ray spectroscopy
EIL: Electron injection layer
E-J: Current efficiency-current density
EL: Electroluminescence
ETL: Electron-transporting layer
FTO: Fluorine-doped tin oxide
HIL: Hole injection layer
HTL: Hole-transporting layer
ITO: Indium tin oxide
MEH-PPV: Poly(2-methoxy-5-(2′ethylhexyloxy)-1,4-phenylenevinylene)
OLEDs: Organic light-emitting devices
PEDOT:PSS: Poly(3,4-ethylenedioxythiophene): poly(styrene sulfonate)
SEM: Scanning electron microscopy
XRD: X-ray diffraction
